# Quantitative proteomics analysis to assess protein expression levels in the ovaries of pubescent goats

**DOI:** 10.1186/s12864-022-08699-y

**Published:** 2022-07-13

**Authors:** Ping Qin, Jing Ye, Xinbao Gong, Xu Yan, Maosen Lin, Tao Lin, Tong Liu, Hailing Li, Xiujuan Wang, Yanyun Zhu, Xiaoqian Li, Ya Liu, Yunsheng Li, Yinghui Ling, Xiaorong Zhang, Fugui Fang

**Affiliations:** 1 Department of Animal Veterinary Science, College of Animal Science and Technology, Anhui Agricultural University, 130 Changjiang West Road, Hefei, 230036 Anhui China; 2 Anhui Province Key Laboratory of Local Livestock and Poultry, Genetical Resource Conservation and Breeding, College of Animal Science and Technology, Anhui Agricultural University, Hefei, 230036 Anhui China; 3 Animal Husbandry Development Center, Huoqiu Animal Health Supervision Institute, Huoqiu County, Auditorium Road, Luan, 237400 Anhui China

**Keywords:** Proteomics, iTRAQ, LC–MS/MS, Pubertal onset, Differentially abundant proteins, PPI network, Anhui white goats

## Abstract

**Background:**

Changes in the abundance of ovarian proteins play a key role in the regulation of reproduction. However, to date, no studies have investigated such changes in pubescent goats. Herein we applied isobaric tags for relative and absolute quantitation (iTRAQ) and liquid chromatography–tandem mass spectrometry to analyze the expression levels of ovarian proteins in pre-pubertal (*n* = 3) and pubertal (*n* = 3) goats.

**Results:**

Overall, 7,550 proteins were recognized; 301 (176 up- and 125 downregulated) were identified as differentially abundant proteins (DAPs). Five DAPs were randomly selected for expression level validation by Western blotting; the results of Western blotting and iTRAQ analysis were consistent. Kyoto Encyclopedia of Genes and Genomes pathway enrichment analysis indicated that DAPs were enriched in olfactory transduction, glutathione metabolism, and calcium signaling pathways. Besides, gene ontology functional enrichment analysis revealed that several DAPs enriched in biological processes were associated with cellular process, biological regulation, metabolic process, and response to stimulus. Protein–protein interaction network showed that proteins interacting with CDK1, HSPA1A, and UCK2 were the most abundant.

**Conclusions:**

We identified 301 DAPs, which were enriched in olfactory transduction, glutathione metabolism, and calcium signaling pathways, suggesting the involvement of these processes in the onset of puberty. Further studies are warranted to more comprehensively explore the function of the identified DAPs and aforementioned signaling pathways to gain novel, deeper insights into the mechanisms underlying the onset of puberty.

**Supplementary Information:**

The online version contains supplementary material available at 10.1186/s12864-022-08699-y.

## Background

Puberty is the transitional period between the juvenile state and adulthood; during this phase, animals gain reproductive capacity, which is crucial for their growth and development [1]. In female animals, the hypothalamic–pituitary–ovarian axis regulates puberty and reproductive function [2, 3], and the ovary is the final target of this axis. The physiological activity of the ovary plays a crucial role in regulating the growth and development of animals [4]. Gonadotropin-releasing hormone (GnRH) neurons in the arcuate nucleus of the hypothalamus synthesize and secrete GnRH, and during the onset of puberty, their activity and consequently GnRH secretion are increased, which is characterized by pulsed GnRH release [5]. GnRH stimulates gonadotropic cells in the anterior pituitary gland to secrete follicle-stimulating hormone and luteinizing hormone. These hormones act on the ovaries via blood circulation to facilitate the secretion of sex hormones, thereby promoting the rapid development of reproductive organs as well as sexual characteristics and playing a pivotal role in the onset of animal puberty [6, 7]. Besides, the ovaries in turn regulate the hypothalamic–pituitary–ovarian axis by positive and negative feedback via the secreted sex hormones, further affecting puberty [8].

The mechanism underlying the onset of puberty is complex and reportedly involves several factors, including neuroendocrinological, genetic, and environmental factors [9]. The specific regulatory mechanism nevertheless remains unclear. Nguyen et al*.* performed adipose tissue proteomic analyses to study puberty in Brahman heifers; 51 significantly differentially abundant proteins (DAPs) were identified between pre- and post-pubertal heifers. These DAPs were enriched in estrogen signaling and PI3K–Akt signaling pathways, which are known integrators of metabolism and reproduction [10]. Further, Fortes et al*.* investigated how the uterine tissue and its secretion changes in relation to puberty in Brahman heifers. Using a combination of proteomics and transcriptomics, they identified 258 DAPs in the uterine fluid of pre- and post-pubertal cows [11]. Similarly, Ye et al*.* applied proteomics to examine the hypothalamus of pre-pubertal and pubertal female goats, which led to the identification of 69 DAPs. These proteins were enriched in the MAPK, RAS, and PI3K–Akt–mTOR signaling pathways, indicating that the identified DAPs and their related signaling pathways are crucial in regulating puberty in goats [12]. Collectively, the results of such studies indicate that proteomics can be effectively used to assess the hypothalamus as well as other tissues to explore the mechanisms underlying puberty.

Although the expression of retinol-binding protein 4 in the ovaries of female mice has been found to remain unchanged until puberty, a significant increase was noted during puberty [13]; moreover, retinol-binding protein 4 evidently plays a chief role in the transportation and storage of cells in follicular fluid [14]. Tahir et al*.* used whole ovaries of six pre- and six post-pubertal Brahman heifers to perform differential abundance analyses of protein profiles between the two physiological states, identifying 32 steroidogenesis-associated DAPs [15]. An increase in steroidogenesis is observed during puberty [16]. A study identified 36 proteins in ovarian follicular fluid of goats at different developmental stages [17]. Overall, these findings suggest that ovarian proteins play a fundamental role in pubertal development in animals.

**Fig. 1:**
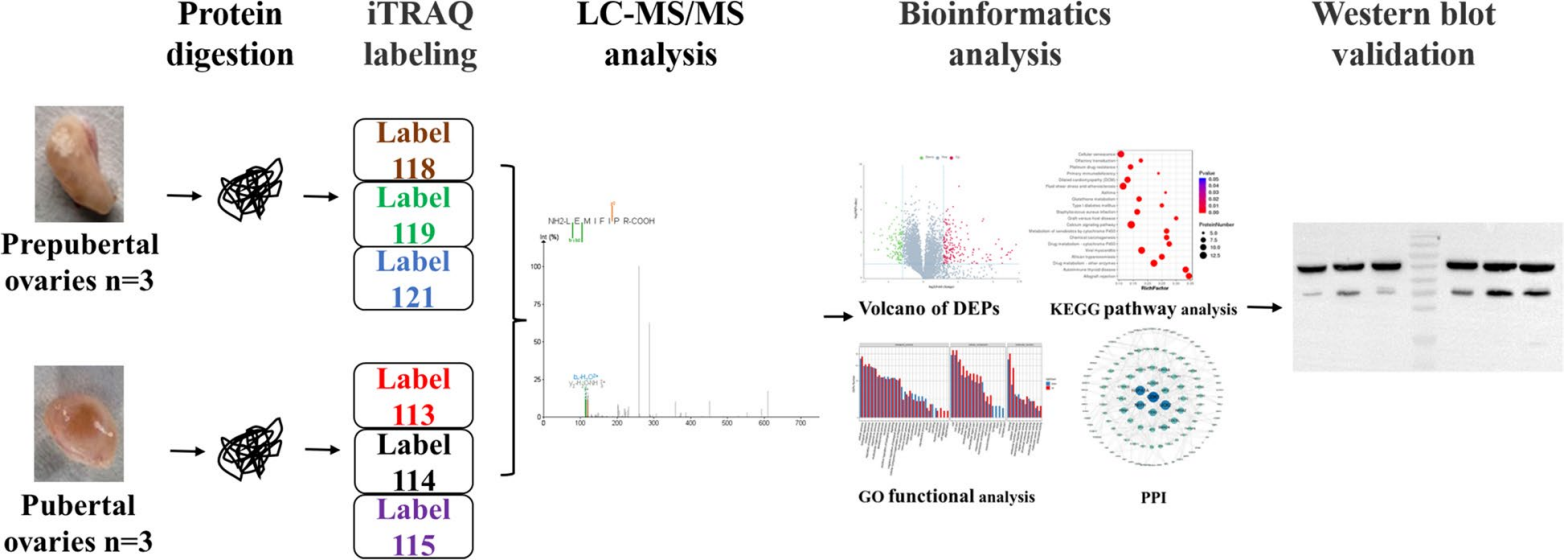
Experimental design and workflow to identify differentially abundant proteins (DAPs) in pre-pubertal (*n* = 3) and pubertal (*n* = 3) goat ovaries

Goats play an important socioeconomic role in the lives of people considering that they are a source of, for example, milk and meat. Puberty is a pivotal stage in female animal development; it marks the first occurrence of ovulation and the onset of reproductive capability [18]. A study reported that lncRNA is related to ovarian steroid hormone synthesis, oogenesis, and oocyte maturation during the growth and development of goats [19]; moreover, the levels of hormones in goats have been found to significantly change during puberty [20]. However, to date, no studies have used proteomics to assess changes in the expression level of proteins in the ovaries of pre-pubertal and pubertal goats. Herein we thus aimed to identify proteins and pathways associated with the onset of puberty by measuring protein abundance levels in the ovaries of prepubertal and pubertal goats. Figure 1 shows the experimental design and workflow. We believe our results should enhance our understanding of the key factors and mechanisms that regulate puberty in goats.

## Results

### Protein Identification

**Fig. 2:**
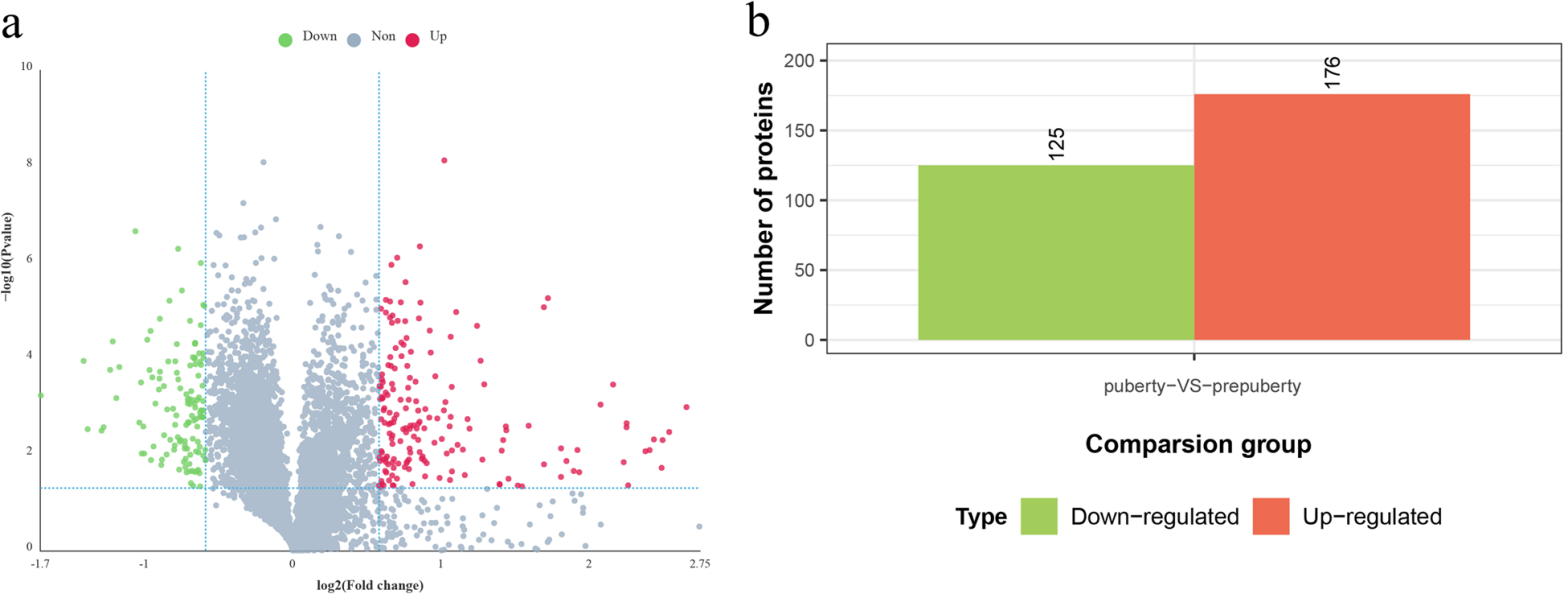
DAP screening. **a** Volcano map. Red and green dots indicate up- and downregulation of protein expression levels, respectively. Gray dots indicate DAPs with insignificant changes in expression. **b** Number of DAPs. In total, 301 DAPs were identified. Orange and green columns represent the number of up- (*n* = 176) and downregulated (*n* = 125) proteins, respectively

Overall, 7,550 proteins were identified and quantified by isobaric tags for relative and absolute quantitation (iTRAQ) proteomics, of which 2,623 were uncharacterized proteins and 4,927 were proteins with known functions. In total, 301 DAPs were identified: 176 of them were up- and 125 were downregulated (Fig. 2a, b). On comparing data pertaining to pubertal with pre-pubertal goat ovaries, MHC class II antigen showed the highest relative upregulation, while DUF4537 domain-containing protein showed the highest relative downregulation. Tables 1 and 2 show the top 15 up- and downregulated proteins, respectively, in pubertal ovaries compared with pre-pubertal ones.

**Table 1:** Top 15 upregulated proteins in pubertal compared to pre-pubertal goat ovaries

Accession	Gene Name	Description	Coverage [%]	Unique Peptides	*P*	Mean_Ratio
Q6BCN2	Cahi-DQA1	MHC class II antigen (Fragment)	0.207	1	0.0010	6.3115
A0A068B4V9	GSTA3	Glutathione S-transferase	0.414	1	0.0034	5.8213
A0A452F8N3	TET3	Methylcytosine dioxygenase TET	0.018	1	0.0050	5.6453
A0A452FL66		Ig-like domain-containing protein	0.155	2	0.0189	5.6192
A0A452F0V7	USP42	USP domain-containing protein	0.006	1	0.0049	5.4207
A0A452FRG5	LMNTD2	LTD domain-containing protein	0.021	1	0.0081	5.3130
P0CH26	HBAII	II alpha globin	0.627	1	0.0086	5.2103
A0A452EDD0	NR5A2	Uncharacterized protein	0.018	1	0.0440	4.8056
A0A452DRV6	ZNF529	Uncharacterized protein	0.014	1	0.0022	4.7709
I6TE27		KIAA1239 (Fragment)	0.017	1	0.0027	4.7686
A0A452F5F2	SERPINI2	SERPIN domain-containing protein	0.044	1	0.0145	4.7063
A0A452DS42		IGv domain-containing protein	0.065	1	0.0003	4.4793
A0A452EDE4	KMT2C	[Histone H3]-lysine (4) N-trimethyltransferase	0.002	1	0.0009	4.2239
A0A452FX60	LOC102185917	Ig-like domain-containing protein	0.147	1	0.0234	3.8227
A0A452FZ42	CCNE1	Cyclin N-terminal domain-containing protein	0.019	1	0.0081	3.7908

**Table 2:** Top 15 downregulated proteins in pubertal compared to pre-pubertal goat ovaries

Accession	Gene Name	Description	Coverage [%]	Unique Peptides	*P*	Mean_Ratio
A0A452GA12	C11orf16	DUF4537 domain-containing protein	0.015	1	0.0006	0.3087
A0A452FKP1	EFCAB6	Uncharacterized protein	0.004	1	0.0001	0.3770
A0A452E9S9	CNMD	BRICHOS domain-containing protein	0.015	1	0.0030	0.3841
P02082		Hemoglobin fetal subunit beta	0.855	10	0.0032	0.4100
A0A452FUY5	PER2	Uncharacterized protein	0.006	1	0.0027	0.4138
A0A452E681	ZP2	ZP domain-containing protein	0.049	4	0.0002	0.4267
A0A452G903		Metallothionein	0.328	1	0.0000	0.4319
A0A452FKU4	ZP3	Zona pellucida sperm-binding protein 3	0.229	9	0.0007	0.4391
A0A452FFM6		G_PROTEIN_RECEP_F1_2 domain-containing protein	0.025	1	0.0002	0.4455
A0A452DYI9		FLYWCH family member 2	0.052	1	0.0000	0.4803
A0A452FEE2		Ig-like domain-containing protein	0.215	2	0.0022	0.4892
A0A452G9F8		Uncharacterized protein	0.189	3	0.0003	0.4930
A0A452EAM2		Uncharacterized protein	0.037	1	0.0095	0.4947
A0A452FCF1	LOC108635821	AIG1-type G domain-containing protein	0.196	2	0.0026	0.4983
A0A0H4PMF2		Esco2	0.01	1	0.0096	0.5003

## Validation of protein expression levels by Western Blotting (WB)

**Fig. 3:**
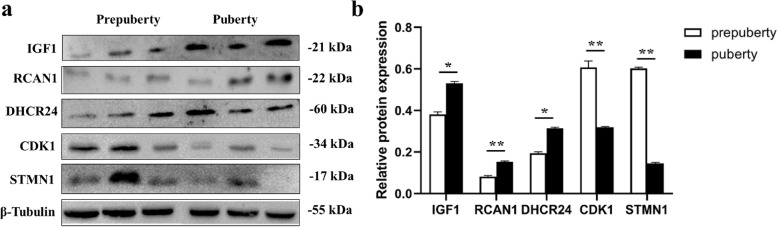
iTRAQ–LC–MS/MS data validation by Western blotting (WB). **a** Abundance of three upregulated [IGF-1 (full-length blots/gels are presented in Additional file 5), RCAN1 (full-length blots/gels are presented in Additional file 6), and DHCR24 (full-length blots/gels are presented in Additional file 7)] and two downregulated [CDK1 (full-length blots/gels are presented in Additional file 8) and STMN1 (full-length blots/gels are presented in Additional file 9)] proteins were analyzed by WB. β-Tubulin (full-length blots/gels are presented in Additional file 8) served as the internal reference. (Some positions of marker were in the middle and some were on the edge of the gels. To display the images more aesthetically, we have cropped them.) **b** Quantitative results for proteins. Values represent mean ± SEM (*n* = 3/group). Statistical significance was assessed using Student’s *t*-test; *P* < 0.05

To confirm the results of iTRAQ–liquid chromatography mass spectrometry (LC–MS)/MS analysis and facilitate subsequent functional study, the abundance of five reproduction-related proteins [upregulated: calcipressin-1 (RCAN1), insulin-like growth factor I (IGF-1), and delta (24)-sterol reductase (DHCR24); downregulated: stathmin (STMN1) and cyclin-dependent kinase 1 (CDK1)] was verified in pubertal and pre-pubertal goat ovaries by WB. WB and iTRAQ–LC–MS/MS analysis data were consistent (Fig. 3a, b), indicating that our proteomics data were highly reliable.

## Cluster analysis of DAPs

**Fig. 4:**
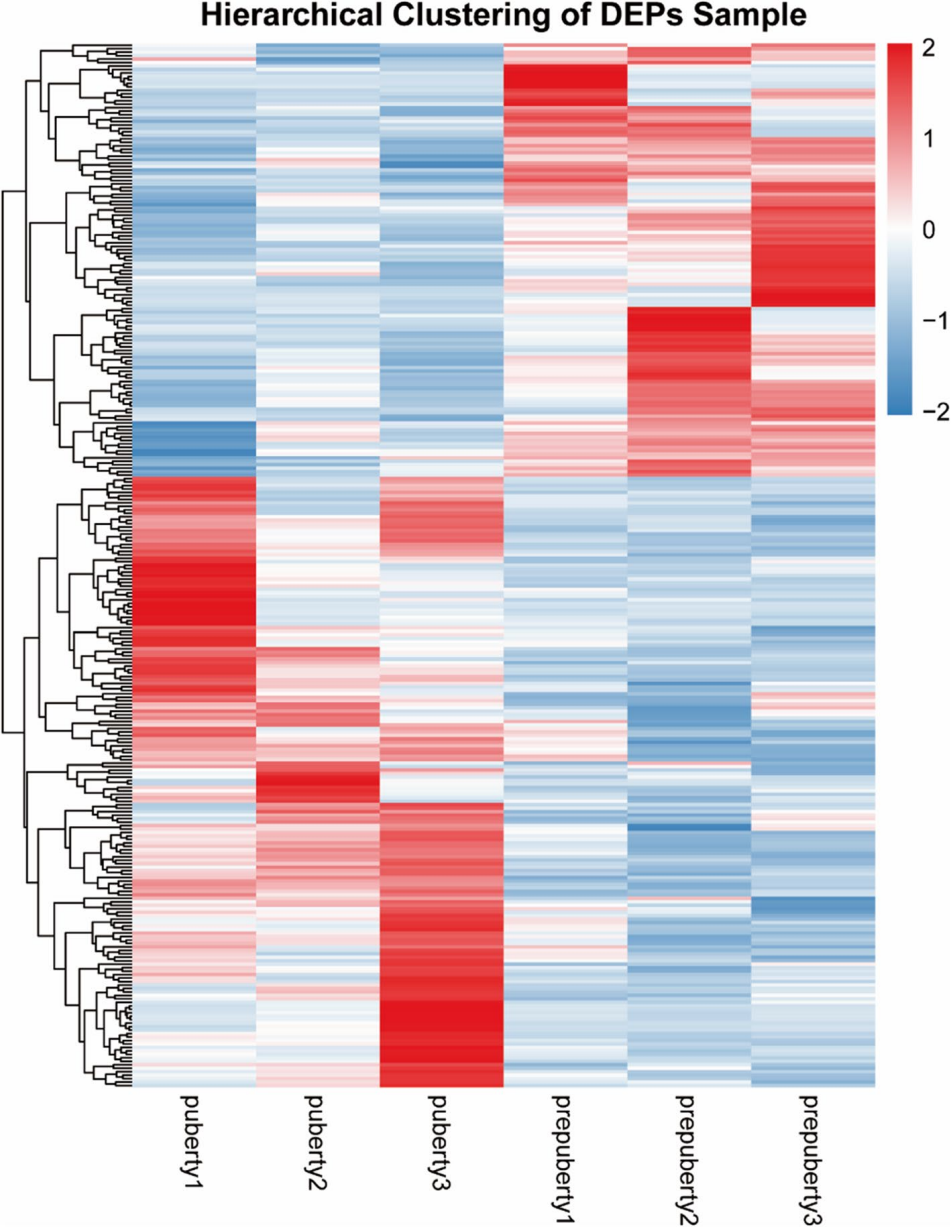
Cluster heatmap showing expression intensity of DAPs. Euclidean distance and hierarchical clustering were used to cluster protein expression patterns. Colors represent relative expression levels of proteins in the ovaries of pre- and pubertal goats, with red and blue representing up- and downregulation, respectively

Euclidean distance and hierarchical clustering were used to cluster DAPs, and dynamic changes in DAPs were compared (Fig. 4). We found significant differences in protein abundance intensity in the ovaries of pubertal and pre-pubertal goats, indicative of differences in protein abundance levels between pubertal and pre-pubertal goat ovaries.

## **Gene Ontology** (GO) Functional Enrichment Analysis.

**Fig. 5:**
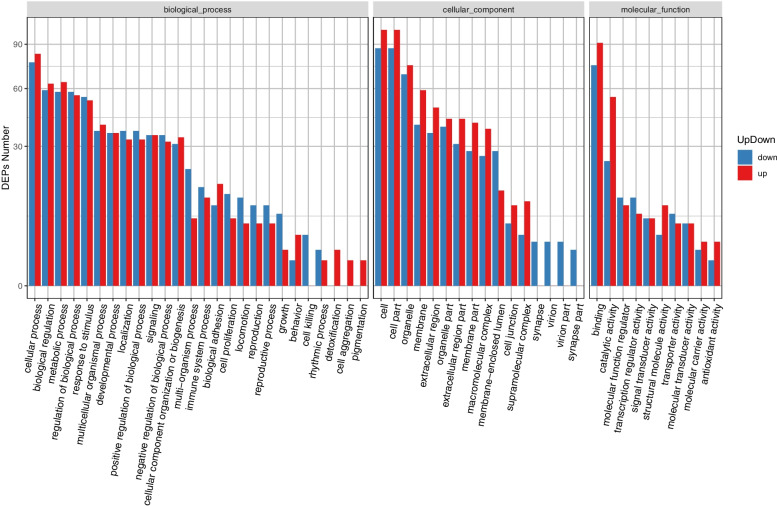
Gene ontology functional analysis of DAPs. *P* < 0.05

To further analyze the functions of the 301 DAPs, they were classified into the following major categories based on their GO annotations: biological process, cellular component, and molecular function. DAPs enriched in biological processes were mainly associated with cellular process, metabolic process, biological regulation, regulation of biological process, and response to stimulus (Fig. 5). Further, the five most abundant terms in the cellular component category were cell part, cell, organelle, membrane, and extracellular region, and those in the molecular function category were binding, catalytic activity, molecular function regulator, transcription regulator activity, and transporter activity.

## Kyoto Encyclopedia of Genes and Genomes (KEGG) pathway enrichment analysis

**Fig. 6:**
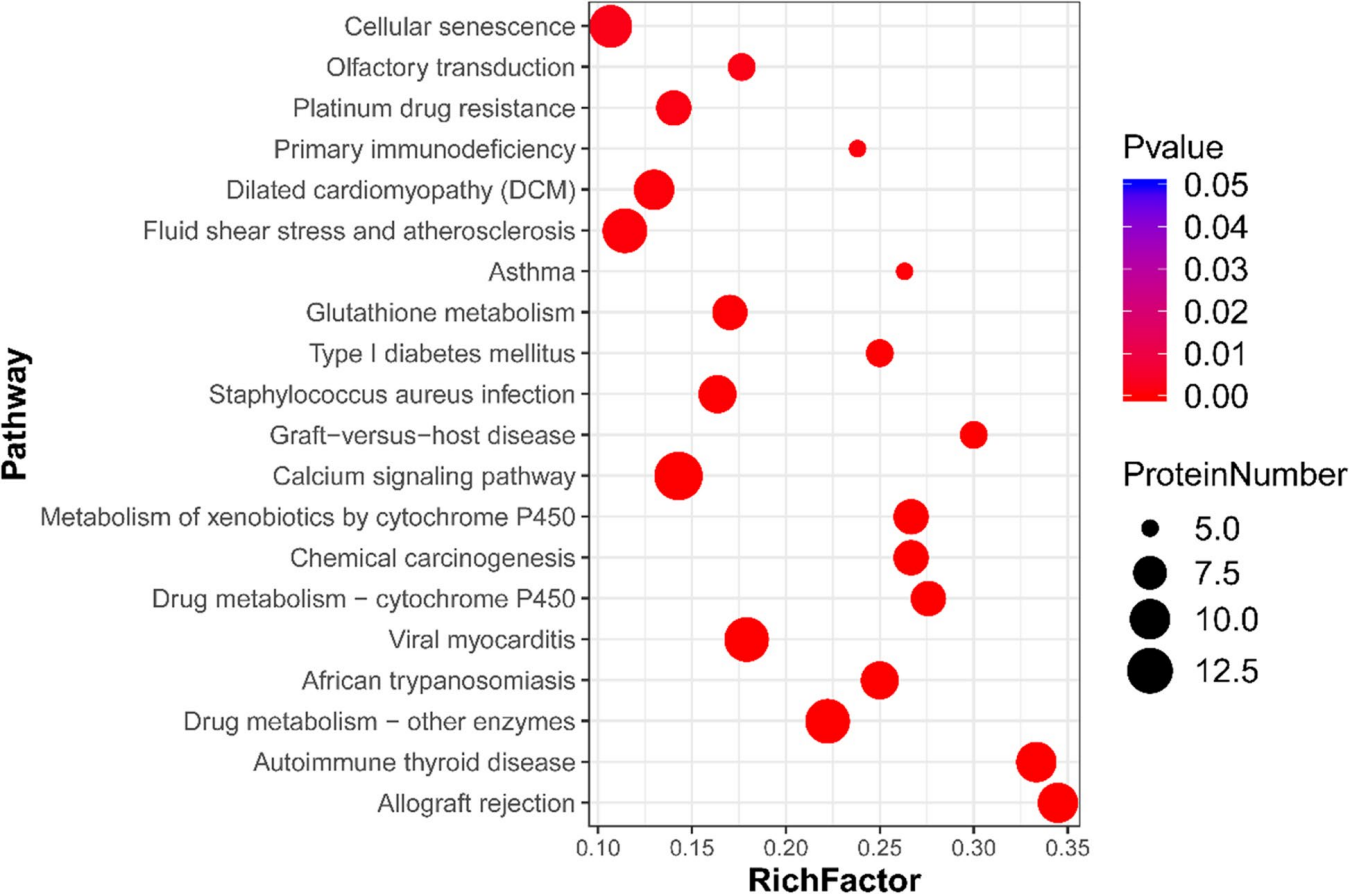
Kyoto Encyclopedia of Genes and Genomes pathway enrichment analysis of DAPs. The larger the bubble, the more the number of DAPs. Bubble color indicates the *P* value; the smaller the *P* value, the higher the level of significance

To further explore the signaling pathways involved in regulating puberty onset in goats, DAPs were subjected to KEGG [21] pathway enrichment analysis (Fig. 6). The 301 DAPs were mapped to 260 KEGG pathways, including glutathione metabolism, olfactory transduction, and calcium signaling pathways, indicating their involvement with pubertal onset in goats.

## Protein–Protein Interaction (PPI) network analysis

**Fig. 7:**
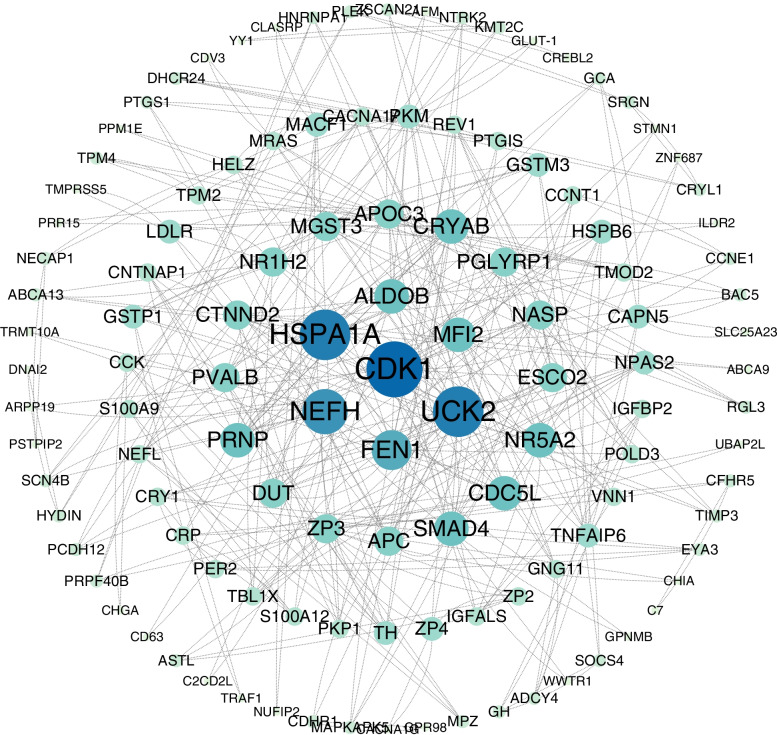
Protein–protein interaction network analysis

The interaction relationships among DAPs in the enriched signaling pathways (KEGG analysis) were found in the STRING database [22], and a PPI network was constructed (Fig. 7), which revealed some key interactions among DAPs. In the PPI network, nodes represent proteins and edges denote predicted functional associations; 222 nodes and 192 edges were found to be interconnected. CDK1, recombinant heat shock 70 kDa protein 1A (HSPA1A), and uridine cytidine kinase 2 (UCK2) occupied the central position in the PPI network and were observed to interact with other DAPs as a hub.

## Discussion

Puberty is a phase of anatomical and physiological development, leading to sexual maturity and reproductive capacity [23]; however, the specific mechanisms underlying puberty remain unclear. Proteins are the material basis of life [24], so changes in the abundance of ovarian proteins are bound to affect pubertal onset. Therefore, we herein performed iTRAQ–LC–MS/MS analysis to compare the expression levels of ovarian proteins in pre-pubertal (*n* = 3) and pubertal (*n* = 3) goats so as to identify core proteins involved in puberty regulation. This led to the identification of 7,550 proteins, of which 301 DAPs met the screening standard.

Among the DAPs identified in this study, MHC class II antigen (fragment) was the most upregulated one in the ovaries of pubertal goats. MHC class II molecules are encoded by the MHC gene locus, which is located on chromosome 6 in humans and chromosome 17 in mice [25]. These molecules play a key regulatory role in several immune responses and are involved in most autoimmune diseases [26]. In addition, MHC genes reportedly influence mating preferences and odor in house mice [27] and even humans [28]. We believe that MHC class II antigen (fragment) affects puberty onset in goats by causing alterations in odor. RCAN1, previously known as DSCR1, was one of the most significantly upregulated proteins in this study. As it is located on human chromosome 21, RCAN1 has been postulated to contribute to mental retardation in Down syndrome. It is evidently involved in transcriptional regulation and/or signal transduction [29]; further, it is expressed in neurons and participates in local protein synthesis during neuronal stimulation [30]. Site-specific phosphorylation of RCAN1 has been reported to regulate bidirectional synaptic plasticity [31]. The downregulation of RCAN1 expression during radial migration of cortical neurons in rats has been observed to impair neural progenitor cell proliferation, resulting in radial migration defects [32]. Besides, both in vitro and in vivo studies have shown that RCAN1 overexpression promotes neuronal loss [33, 34]. Nakata et al*.* found that growth hormone (GH) administration induced RCAN1 mRNA expression in the liver of hypophysectomized rats [35]. It is a known fact that the secretion rate and amount of GH increase during puberty [36], and in vitro experiment results have indicated that GH directly stimulates GnRH secretion from GT1-7 cells [37]. Therefore, it seems that RCAN1 indirectly regulates puberty onset via GH.

In this study, IGF-1 expression levels were also significantly upregulated in the ovaries of goats at puberty. IGF-1, a polypeptide hormone, is an important modulator of GH activity, and it plays a pivotal role in cell differentiation and proliferation [38] as well as in bone growth and development [39]. In addition, in the developing central nervous system, IGF-1 mediates changes in morphology, synaptic efficacy, and cellular organization, enabling cells of the central nervous system to respond to continuous external and internal stimuli [40]. IGF-1 is reportedly the upstream regulator of *KiSS-1* [41]; moreover, IGF-1 is an important factor in reproductive and neuroendocrine function regulation, and it also participates in GnRH synthesis and release [42]. Intracerebroventricular administration of IGF-1 has been reported to promote GnRH secretion by activating *KiSS-1* expression in the brain of pre-pubertal female rats, advancing the onset of puberty [43]. According to our findings, we believe that this phenomenon could be due to IGF-1 changing the sensitivity of KiSS-1 neurons to stimuli or changing the intensity and duration of neuronal synaptic activity. Further studies are warranted to explore the roles of RCAN1 and IGF-1 in the onset of puberty in goats.

Herein the enrichment of DAPs in olfactory transition, glutathione metabolism, and calcium signaling pathways suggested the involvement of these pathways in the onset of puberty in goats. Olfactory signals evidently play a key role in the onset of mammalian puberty [44]. The mammalian olfactory system can effectively detect pheromones; externally released steroids in mammals have been reported to function as pheromones and provide direct information pertaining to internal hormone state [45]. Olfactory sensory neurons are the first order neurons in the olfactory system and the initial site of odor detection [46]; the signals generated are transmitted to the hypothalamus, which affect reproduction as well as fertility [47]. Several pheromones affect pubertal onset in animals [48]. The olfactory system influences the timing of puberty in mice via GnRH neurons [49]. Furthermore, male scents can reportedly accelerate the onset of puberty in females, promoting uterine growth and the first estrus [50]. Glutathione is a ubiquitous tripeptide composed of glutamic acid, cysteine, and glycine, and it plays a chief role in cell proliferation regulation [51, 52]. Glutamate–cysteine ligase is the rate-limiting step in glutathione synthesis [53]. Glutamate dehydrogenase catalyzes glutamate synthesis; a study reported the hypothalamic content of glutamate to significantly increase in female rats at first proestrus [54], which may be related to the synthesis of glutathione, considering that the content of glutathione reaches its peak during this time [55]. In addition, subcutaneous administration of glutathione has been found to predate the onset of puberty in immature female rats, and the weight of the pituitary gland and ovary was found to significantly increase [56]. Similarly, intracerebroventricular administration of glutathione was reported to increase plasma levels of follicle-stimulating hormone [57], which is known to play a fundamental role in puberty [58]. From an overall perspective, it appears that glutathione and its metabolism play a chief role in regulating the onset of puberty.

Luteinizing hormone reportedly acts via protein kinase A and C to modulate T-type calcium currents and intracellular calcium transients in mice Leydig cells [59]. Calcium, a critical intracellular messenger, regulates numerous signal transduction pathways and is associated with steroid production [60]. In the excitable cells of the nervous system, an increase in free calcium levels evidently initiates neurotransmitter release [61], which consequently affects the onset of puberty in animals [62, 63]. In this study, the expression level of phosphodiesterase (PDE) in the calcium signaling pathway was significantly upregulated. It is notable that the onset of puberty corresponds to the maturation of oocytes [64]. In most mammals, oocytes enter the early stage of meiosis during the fetal stage and their development then stops until ovulation or atresia [65]. The preovulatory gonadotropin surge leads to a decrease in cyclic guanosine monophosphate levels in oocytes and an increase in intracellular PDE activity, which leads to a decrease in cyclic adenosine monophosphate (cAMP) levels and recovery of meiosis [66, 67]. PDE activity regulation is a complex, important approach to control the level of intracellular second messenger cAMP [68]. In addition, gonadotropins activate adenylyl cyclase in oocytes and facilitate its interaction with G protein to catalyze the conversion of adenosine triphosphate to cAMP [69, 70]. In mammalian oocytes, cAMP maintains meiotic arrest by inhibiting maturation-promoting factor activation and stimulating cAMP-dependent protein kinase A [71]. This prevents oocytes from resuming meiosis prematurely, and they thus obtain normal development ability [72]. Herein we found that PDE and adenylate cyclase type 4 expression levels were significantly upregulated in goat ovaries during puberty. This could be a method to regulate intracellular cAMP levels so that the meiosis of oocytes and eventually the onset of puberty can occur normally.

Our PPI network revealed that proteins interacting with CDK1, HSPA1A, and UCK2 were the most abundant. While HSPA1A and UCK2 have been discussed in the context of diseases such as cancer [73, 74], their involvement in puberty remains to be reported. CDK1 is a cell cycle-related enzyme that drives cell division and is the main regulator of the cell cycle process [75, 76]. In addition, mammalian cells express other cyclin kinases and phosphorylate cellular proteins to drive the cell cycle process [77, 78]. Intriguingly, CDK1 can replace other CDKs and is sufficient to drive the mammalian cell cycle [79]. Artificial knockout of *CDK1* has been found to lead to early embryo death, and conditional knockout of *CDK1* in viable mice causes cell proliferation disorder [80]. Besides, CDK1 is essential and sufficient to drive the resumption of meiosis in mouse oocytes, and the process of meiosis is regulated by CDK1 activity [81, 82]. In combination with the results of these studies, our findings imply that CDK1 plays a prominent role in mammalian reproduction.

## Conclusions

To summarize, 301 DAPs were identified on analyzing ovarian protein expression levels in pubertal and pre-pubertal goats. We believe that the DAPs identified herein directly or indirectly regulate pubertal onset. Besides, olfactory conduction, glutathione metabolism, and calcium signaling pathways seem to play a significant role in the onset of puberty in goats. Further studies are nevertheless warranted to explore the specific molecular mechanisms underlying the onset of puberty.

## Materials and methods

### Experimental Design.

Quantitative proteomics techniques based on iTRAQ were used to identify and characterize DAPs between prepubertal and pubertal goats. Goat ovaries were collected and suspended in protein lysate; ovarian proteins were then enzymatically digested with trypsin to obtain peptides. The hydrolyzed peptides were subsequently labeled with iTRAQ labels. Peptide separation and high-performance LC were performed, followed by MS and bioinformatics analyses. To verify the accuracy of our proteomics data, the expression levels of several proteins were assessed by WB.

## Ovary collection

This study was authorized and endorsed by the Animal Care and Use Committee of Anhui Agricultural University. All experimental procedures involving goats were performed according to the Regulations for the Administration of Affairs Concerning Experimental Animals (Ministry of Science and Technology, China; revised in June 2004). Three pre-pubertal (2.5 months of age, 8.1 ± 0.3 kg) and three pubertal (4.5–5.0 months of age, 20.16 ± 0.35 kg) Anhui white goats (an indigenous Chinese breed) were housed under the same conditions on a farm in Feixi County, Anhui Province, China. The date of puberty onset in female goats was defined as the date of the first estrus detected by male goats and by monitoring changes in the appearance of the vulva [83]. Rams test conditions were performed twice daily at 08:00 and 16:00. The cunnus of pubescent goats was inflamed and mature follicles were observed in the ovaries. The animals were deeply anesthetized by intramuscularly injecting 1% pentobarbital (60 mg/kg, Solarbio, P8410, China). The left ovary was then surgically removed and immediately frozen in liquid nitrogen, and they were stored at − 80 °C until needed.

## Protein extraction

The ovaries were transferred into six 5-mL centrifuge tubes. An appropriate amount of 1 × cocktail was then added, followed by the addition of Ethylene Diamine Tetraacetic Acid (Ameresco, Shanghai, China) and sodium dodecyl sulfate (SDS, Solarbio, Beijing, China) lysis buffer 3. Finally, two 5-mm magnetic beads were added to the centrifuge tubes with tweezers to enable thorough grinding. After incubation on ice for 5 min, a tissue lyser (JXFSTPRP, Shanghai, China) was used to break and lyse the ovaries. The samples were then centrifuged at 25,000 × *g* and 4 °C for 15 min. To the supernatant thus obtained, dithiothreitol (Bio-Rad, Hercules, CA, USA) was added at a final concentration of 10 mM, followed by incubation in a water bath at 56 °C for 1 h. Subsequently, iodoacetamide (Sigma-Aldrich, St. Louis, MO, USA) was added at a final concentration of 55 mM, and the suspension was incubated in the dark for 45 min. Five times the volume of precooled acetone (Lingfeng, Shanghai, China) was then added, followed by incubation at − 20 °C for 2 h and centrifugation at 25,000 × *g* and 4 °C for 15 min. The supernatant thus obtained was discarded, and a suitable amount of lysis buffer 3 without SDS was added to the precipitate to promote protein dissolution through a tissue lyser. After centrifugation at 25,000 × *g* and 4 °C for 15 min, the supernatant was obtained, which represented the protein extract.

## Proteolysis and peptide labeling

To a 1.5-mL centrifuge tube, 100 μg protein was added. As per trypsin (μg):substrate protein (μg) ratio of 1:20, an enzyme solution was then added, and the solution was vortexed and then centrifuged at low speed for 1 min, followed by incubation at 37 °C for 4 h. The digested peptide solution was then subjected to desalination and freeze-dried.

Peptides were reconstituted in 0.5 M tetraethylammonium bromide and processed with 8-plex iTRAQ reagent (Applied Biosystems), according to manufacturer instructions. Briefly, iTRAQ labeling reagents were thawed to room temperature, and 50 μL isopropyl alcohol was added to each reagent tube. The tube was then vortexed and shaken at low speed. Samples were labeled as follows: sample puberty 1 (113 tags), 2 (114 tags), and 3 (115 tags) and sample prepuberty 1 (118 tags), 2 (119 tags), and 3 (121 tags). After labeling, all samples were incubated at room temperature for 2 h. The labeled peptide mixtures were then pooled and dried by vacuum centrifugation.

## Peptide Separation and High-Performance Liquid Chromatography.

The LC-20AB liquid phase system (Shimadzu, Kyoto, Japan) was used for liquid phase separation of samples. We used a Gemini C18 separation column (4.6 × 250 mm, 5 μm). iTRAQ-labeled peptide mixtures were reconstituted in mobile phase A (5% acetonitrile, pH 9.8) and injected; gradient elution was used at a flow rate of 1 mL/min: 5% mobile phase B (95% acetonitrile, pH 9.8) for 10 min, 5%–35% mobile phase B for 40 min, 35%–95% mobile phase B for 1 min, mobile phase B for 3 min, and 5% mobile phase B for 10 min. Elution was monitored by measuring absorbance at 214 nm, and fractions were collected every 1 min. The eluted peptides were pooled into 20 fractions, followed by freeze drying.

The separation was performed using the Easy-nLC™ 1200 system (Thermo Fisher Scientific, San Jose, CA). The freeze-dried peptide samples were reconstituted in mobile phase A (2% acetonitrile and 0.1% formic acid), centrifuged at 20,000 × *g* for 10 min, and the supernatant thus obtained was collected. The samples then entered a tandem self-packed C18 column (internal diameter, 75 μm; column size, 1.9 μm; column length, 25 cm) and were separated at a flow rate of 200 nL/min via the following gradient: 0–3 min, 5% mobile phase B (80% acetonitrile, 0.1% formic acid); 3–45 min, linear increase of mobile phase B from 8 to 44%; 45–50 min, linear increase of mobile phase B from 44 to 60%; 50–53 min, linear increase of mobile phase B from 60 to 100%; and 53–60 min, 80% mobile phase B. The nanoliter liquid phase separation end was directly connected to the mass spectrometer.

## Mass spectrometry

The peptides separated in liquid phase were ionized by the nano-ESI source and then transferred to an Orbitrap Exploris 480 (Thermo Fisher Scientific, San Jose, CA) tandem mass spectrometer in data-dependent acquisition mode. The main settings were as follows: ion source voltage, 2.1 kV; scanning range of primary MS, 350–1,600 m*/z* with resolution of 60,000; and initial *m/z* of secondary MS, 100 m*/z* with resolution of 15,000. The screening conditions for the parent ions of secondary fragmentation were as follows: the charge was from 2 + to 7 + , and the peak strength was greater than 50,000 in the first 1 s. The ion fragmentation mode was higher energy collision-induced dissociation; fragment ions were detected in the Orbitrap, and the dynamic exclusion time was set at 30 s. The auto gain control was set to Level 1 1E6 and Level 2 1E5.

## DAPs screening

The automated software IQuant [84], which integrates Mascot Percolator [85] algorithm, was used quantitatively analyze peptide samples labeled with isobaric tags. To assess the confidence of peptides, peptide-spectrum matches were pre-filtered at a false discovery rate (FDR) of 1%. Subsequently, based on the “parsimony principle,” identified peptide sequences were assembled into a set of confident proteins. To control the rate of false positives at the protein level, a protein FDR at 1%, which is based on Picked protein FDR strategy [86], was also estimated after protein inference (protein-level FDR ≤ 0.01). When the ratio of pubertal ovaries compared with pre-pubertal ovaries was 1.5 or 0.67, proteins were considered to up- or downregulated.

## Western blot

Samples were obtained from the same ovaries as those used for iTRAQ–LC–MS/MS analysis, and protein concentration was determined using the BCA protein detection kit. After adding a protein loading buffer, the sample was boiled for 10 min at 100℃. Subsequently, 20 μg protein was subjected to SDS–polyacrylamide gel electrophoresis (Bio-Rad, Hercules, CA, USA), and protein bands thus obtained were transferred to a polyvinylidene fluoride (Merck Millipore, Billerica, MA, USA) membrane. The protein was sealed at room temperature for 2 h with 5% skimmed milk powder and then incubated with each of the following antibodies for overnight at 4℃: rabbit anti-RCAN1 (1:500, T73889, Abmart), rabbit anti-IGF1 (1:500, PA3635, Abmart), rabbit anti-DHCR24 (1:1000, TD12472, Abmart), rabbit anti-STMN1 (1:500, PA4263, Abmart), and rabbit anti-CDK1 (1:500, T55176, Abmart). The membranes were washed thrice with Tris-buffered saline with Tween (BioSharp, Hefei, China) for 10 min each time, and then a secondary antibody, i.e., horseradish peroxidase-labeled goat anti-rabbit immunoglobulin G (1:3,000, GB23303, Servicebio), was added, followed by incubation at room temperature for 1 h. After washing thrice with Tris-buffered saline with Tween, the membrane was finally analyzed using a chemiluminescence imaging analyzer. The test strips were excised, and their gray value was analyzed by the image analysis software ImageJ. β-Tubulin (1:2,500; Proteintech, Wuhan, China) was used as the internal control; the relative gray value of target proteins was obtained using the ratio of the gray values of target proteins and β-tubulin.

## Bioinformatics

Hierarchical Cluster [87] and Java TreeView [88] were used for hierarchical clustering analysis. All genes corresponding to DAPs were subjected to GO annotation [89] and KEGG [90] pathway analyses. Gene symbols of DAPs were submitted to the STRING database [91], and Cytoscape v3.2.1 [92] was used to generate and analyze PPI networks.

## Statistical analysis

GraphPad Prism 8 (GraphPad Software, San Diego, CA) was used for histogram plotting. Values represent mean ± SEM, and Student’s *t-*test was used to compare relative expression levels of ovarian proteins between pubertal and pre-pubertal goats. IBM SPSS Statistics 26 (SPSS Inc., Chicago, USA) was used for data analysis. *P* < 0.05 indicates statistical significance.

## Statement

This study was authorized and endorsed by the Animal Care and Use Committee of Anhui Agricultural University and reported in accordance with the ARRIVE guidelines. All experimental procedures involving goats were performed according to the Regulations for the Administration of Affairs Concerning Experimental Animals (Ministry of Science and Technology, China; revised in June 2004).

## Supplementary Information

**Additional file** **1.**

**Additional file** **2.**

**Additional file** **3.**

**Additional file** **4.**

**Additional file** **5.**

## Data Availability

The mass spectrometry proteomics data have been deposited to the ProteomeXchange Consortium via the PRIDE [93] partner repository with the dataset identifier PXD032665.

## Abbreviations

iTRAQ: Isobaric tags for relative and absolute quantitation
DAPs: Differentially abundant proteins
GnRH: Gonadotropin-releasing hormone
MS: Mass spectrometry
WB: Western blotting
SDS: Sodium dodecyl sulfate
FDR: False discovery rate
GO: Gene ontology
KEGG: Kyoto Encyclopedia of Genes and Genomes
PPI: Protein–protein interaction
RCAN1: Calcipressin-1
IGF-1: Insulin-like growth factor I
DHCR24: Delta (24)-sterol reductase
STMN1: stathmin
CDK1: Cyclin-dependent kinase 1
HSPA1A: Recombinant heat shock 70 kDa protein 1A
UCK2: Uridine cytidine kinase 2
GH: Growth hormone
PDE: Phosphodiesterase
cAMP: Cyclic adenosine monophosphate

## References

[1] Parent AS, Teilmann G, Juul A, Skakkebaek NE, Toppari J, Bourguignon JP (2003). The timing of normal puberty and the age limits of sexual precocity: variations around the world, secular trends, and changes after migration. Endocr Rev.

[2] Chen BY (1997). Acupuncture normalizes dysfunction of hypothalamic-pituitary-ovarian axis. Acupunct Electrother Res.

[3] Zhu H, Nan S, Suo C, Zhang Q, Hu M, Chen R, Wan J, Li M, Chen J, Ding M (2019). Electro-Acupuncture Affects the Activity of the Hypothalamic-Pituitary-Ovary Axis in Female Rats. Front Physiol.

[4] Matsuda F, Inoue N, Manabe N, Ohkura S (2012). Follicular growth and atresia in mammalian ovaries: regulation by survival and death of granulosa cells. J Reprod Dev.

[5] Clarke IJ, Cummins JT (1982). The temporal relationship between gonadotropin releasing hormone (GnRH) and luteinizing hormone (LH) secretion in ovariectomized ewes. Endocrinology.

[6] Ramirez VD, Sawyer CH (1965). Advancement of Puberty in the Female Rat by Estrogen. Endocrinology.

[7] Sagodi L, Solyom E, Kiss-Toth E (2018). Neuroendocrine mechanisms controlling the development in puberty. A literature overview. Orv Hetil.

[8] Day ML, Imakawa K, Wolfe PL, Kittok RJ, Kinder JE (1987). Endocrine mechanisms of puberty in heifers. Role of hypothalamo-pituitary estradiol receptors in the negative feedback of estradiol on luteinizing hormone secretion. Biol Reprod.

[9] Brito VN, Latronico AC (2015). Puberty: when is it normal?. Arch Endocrinol Metab.

[10] Nguyen LT, Zacchi LF, Schulz BL, Moore SS, Fortes MRS (2018). Adipose tissue proteomic analyses to study puberty in Brahman heifers. J Anim Sci.

[11] Fortes MRS, Zacchi LF, Nguyen LT, Raidan F, Weller M, Choo JJY, Reverter A, Rego JPA, Boe-Hansen GB, Porto-Neto LR (2018). Pre- and post-puberty expression of genes and proteins in the uterus of Bos indicus heifers: the luteal phase effect post-puberty. Anim Genet.

[12] Ye J, Yan X, Qin P, Gong X, Li H, Liu Y, Yu T, Zhang Y, Ling Y, Cao H (2021). Proteomic analysis of hypothalamus in prepubertal and pubertal female goat. J Proteomics.

[13] Jiang Y, Zhao Y, Chen S, Chen L, Li C, Zhou X (2018). Regulation by FSH of the dynamic expression of retinol-binding protein 4 in the mouse ovary. Reprod Biol Endocrinol.

[14] Kawaguchi R, Zhong M, Kassai M, Ter-Stepanian M, Sun H (2015). Vitamin A Transport Mechanism of the Multitransmembrane Cell-Surface Receptor STRA6. Membranes (Basel).

[15] Tahir MS, Nguyen LT, Schulz BL, Boe-Hansen GA, Thomas MG, Moore SS, Lau LY, Fortes MRS. Proteomics recapitulates ovarian proteins relevant to puberty and fertility in Brahman Heifers (Bos indicus L.). Genes (Basel). 2019;10(11):923. 10.3390/genes10110923 PMC6895798 31726744

[16] Bae YJ, Zeidler R, Baber R, Vogel M, Wirkner K, Loeffler M, Ceglarek U, Kiess W, Korner A, Thiery J (2019). Reference intervals of nine steroid hormones over the life-span analyzed by LC-MS/MS: Effect of age, gender, puberty, and oral contraceptives. J Steroid Biochem Mol Biol.

[17] Paula Junior AR, van Tilburg MF, Lobo MDP, Monteiro-Moreira ACO, Moreira RA, Melo CHS, Souza-Fabjan JMG, Araujo AA, Melo LM, Teixeira DIA (2018). Proteomic analysis of follicular fluid from tropically-adapted goats. Anim Reprod Sci.

[18] Gao X, Ye J, Yang C, Zhang K, Li X, Luo L, Ding J, Li Y, Cao H, Ling Y (2017). Screening and evaluating of long noncoding RNAs in the puberty of goats. BMC Genomics.

[19] Liu Y, Qi B, Xie J, Wu X, Ling Y, Cao X, Kong F, Xin J, Jiang X, Wu Q (2018). Filtered reproductive long non-coding RNAs by genome-wide analyses of goat ovary at different estrus periods. BMC Genomics.

[20] Devrim AK, Elmaz O, Mamak N, Sudagidan M (2015). Levels of hormones and cytokines associated with growth in Honamli and native hair goats. Pol J Vet Sci.

[21] Kanehisa M, Goto S (2000). KEGG: kyoto encyclopedia of genes and genomes. Nucleic Acids Res.

[22] von Mering C, Jensen LJ, Snel B, Hooper SD, Krupp M, Foglierini M, Jouffre N, Huynen MA, Bork P: STRING: known and predicted protein-protein associations, integrated and transferred across organisms. Nucleic Acids Res 2005, 33(Database issue):D433–437. 10.1093/nar/gki005 PMC539959 15608232

[23] Ma T, Zhou Y, Xia Y, Jin H, Wang B, Wu J, Ding J, Wang J, Yang F, Han X (2021). Environmentally relevant perinatal exposure to DBP disturbs testicular development and puberty onset in male mice. Toxicology.

[24] Leonen CJA, Upadhyay E, Chatterjee C (2018). Studies of biochemical crosstalk in chromatin with semisynthetic histones. Curr Opin Chem Biol.

[25] Dilthey A, Cox C, Iqbal Z, Nelson MR, McVean G (2015). Improved genome inference in the MHC using a population reference graph. Nat Genet.

[26] Unanue ER, Turk V, Neefjes J (2016). Variations in MHC Class II Antigen Processing and Presentation in Health and Disease. Annu Rev Immunol.

[27] Penn DJ, Potts WK (1999). The Evolution of Mating Preferences and Major Histocompatibility Complex Genes. Am Nat.

[28] Havlicek J, Winternitz J, Roberts SC (1800). Major histocompatibility complex-associated odour preferences and human mate choice: near and far horizons. Philos Trans R Soc Lond B Biol Sci.

[29] Fuentes JJ, Pritchard MA, Planas AM, Bosch A, Ferrer I, Estivill X (1995). A new human gene from the Down syndrome critical region encodes a proline-rich protein highly expressed in fetal brain and heart. Hum Mol Genet.

[30] Wang W, Zhu JZ, Chang KT, Min KT (2012). DSCR1 interacts with FMRP and is required for spine morphogenesis and local protein synthesis. EMBO J.

[31] Dudilot A, Trillaud-Doppia E, Boehm J: RCAN1 Regulates Bidirectional Synaptic Plasticity. Curr Biol 2020, 30(7):1167–1176 e1162. 10.1016/j.cub.2020.01.041 32084406

[32] Li Y, Wang J, Zhou Y, Li D, Xiong ZQ (2015). Rcan1 deficiency impairs neuronal migration and causes periventricular heterotopia. J Neurosci.

[33] Martin KR, Corlett A, Dubach D, Mustafa T, Coleman HA, Parkington HC, Merson TD, Bourne JA, Porta S, Arbones ML (2012). Over-expression of RCAN1 causes Down syndrome-like hippocampal deficits that alter learning and memory. Hum Mol Genet.

[34] Lee EJ, Lee JY, Seo SR, Chung KC (2007). Overexpression of DSCR1 blocks zinc-induced neuronal cell death through the formation of nuclear aggregates. Mol Cell Neurosci.

[35] Nakata T, Toyoshima Y, Yagi T, Katsumata H, Tokita R, Minami S (2020). Growth hormone increases regulator of calcineurin 1–4 (Rcan1-4) mRNA through c-JUN in rat liver. PLoS ONE.

[36] Rose SR, Municchi G, Barnes KM, Kamp GA, Uriarte MM, Ross JL, Cassorla F, Cutler GB (1991). Spontaneous growth hormone secretion increases during puberty in normal girls and boys. J Clin Endocrinol Metab.

[37] Cannarella R, Paganoni AJJ, Cicolari S, Oleari R, Condorelli RA, La Vignera S, Cariboni A, Calogero AE, Magni P. Anti-Mullerian hormone, growth hormone, and insulin-like growth factor 1 modulate the migratory and secretory patterns of GnRH neurons. Int J Mol Sci. 2021;22(5):2445. 10.3390/ijms22052445 PMC7957759 33671044

[38] Hefka Blahnova V, Dankova J, Rampichova M, Filova E (2020). Combinations of growth factors for human mesenchymal stem cell proliferation and osteogenic differentiation. Bone Joint Res.

[39] d’Angelo DM, Di Donato G, Breda L, Chiarelli F. Growth and puberty in children with juvenile idiopathic arthritis. Pediatr Rheumatol Online J. 2021;19(1):28. 10.1186/s12969-021-00521-5 PMC7953722 33712046

[40] Dyer AH, Vahdatpour C, Sanfeliu A, Tropea D (2016). The role of Insulin-Like Growth Factor 1 (IGF-1) in brain development, maturation and neuroplasticity. Neuroscience.

[41] Hiney JK, Srivastava VK, Les Dees W (2010). Insulin-like growth factor-1 stimulation of hypothalamic KiSS-1 gene expression is mediated by Akt: effect of alcohol. Neuroscience.

[42] Shao P, Wang Y, Zhang M, Wen X, Zhang J, Xu Z, Hu M, Jiang J, Liu T (2019). The interference of DEHP in precocious puberty of females mediated by the hypothalamic IGF-1/PI3K/Akt/mTOR signaling pathway. Ecotoxicol Environ Saf.

[43] Hiney JK, Srivastava VK, Pine MD, Les Dees W (2009). Insulin-like growth factor-I activates KiSS-1 gene expression in the brain of the prepubertal female rat. Endocrinology.

[44] Corona R, Jayakumar P, Carbajo Mata MA, Del Valle-Diaz MF, Luna-Garcia LA, Morales T (2021). Sexually dimorphic effects of prolactin treatment on the onset of puberty and olfactory function in mice. Gen Comp Endocrinol.

[45] Liberles SD (2014). Mammalian pheromones. Annu Rev Physiol.

[46] Keller A, Zhuang H, Chi Q, Vosshall LB, Matsunami H (2007). Genetic variation in a human odorant receptor alters odour perception. Nature.

[47] Yoon H, Enquist LW, Dulac C (2005). Olfactory inputs to hypothalamic neurons controlling reproduction and fertility. Cell.

[48] Novotny MV, Ma W, Wiesler D, Zidek L (1999). Positive identification of the puberty-accelerating pheromone of the house mouse: the volatile ligands associating with the major urinary protein. Proc Biol Sci.

[49] Boehm U, Zou Z, Buck LB (2005). Feedback loops link odor and pheromone signaling with reproduction. Cell.

[50] Vandenbergh JG (1969). Male odor accelerates female sexual maturation in mice. Endocrinology.

[51] Lu SC (2013). Glutathione synthesis. Biochim Biophys Acta.

[52] Pallardo FV, Markovic J, Garcia JL, Vina J (2009). Role of nuclear glutathione as a key regulator of cell proliferation. Mol Aspects Med.

[53] Bachhawat AK, Yadav S (2018). The glutathione cycle: Glutathione metabolism beyond the gamma-glutamyl cycle. IUBMB Life.

[54] Roth CL, McCormack AL, Lomniczi A, Mungenast AE, Ojeda SR (2006). Quantitative proteomics identifies a change in glial glutamate metabolism at the time of female puberty. Mol Cell Endocrinol.

[55] Pasha KV, Vijayan E (1989). Glutathione distribution in rat brain at different ages and the effect of intraventricular glutathione on gonadotropin levels in ovariectomized steroid-primed rats. Brain Res Bull.

[56] Vali Pasha K (2007). Involvement of glutathione in puberty and FSH release. Neurosci Lett.

[57] Pasha KVJH: Indian ETD Repository @ INFLIBNET: Studies on the interaction between glutathione and hypothalamic peptide hormones in the Rat brain.

[58] Recchia K, Jorge AS, Pessoa LVF, Botigelli RC, Zugaib VC, de Souza AF, Martins DDS, Ambrosio CE, Bressan FF, Pieri NCG. Actions and roles of FSH in germinative cells. Int J Mol Sci. 2021;22(18):10110. 10.3390/ijms221810110 PMC8470522 34576272

[59] Costa RR, Reis RI, Aguiar JF, Varanda WA (2011). Luteinizing hormone (LH) acts through PKA and PKC to modulate T-type calcium currents and intracellular calcium transients in mice Leydig cells. Cell Calcium.

[60] Rossato M, Nogara A, Merico M, Ferlin A, Garolla A, Foresta C (2001). Store-operated calcium influx and stimulation of steroidogenesis in rat Leydig cells: role of Ca(2+)-activated K(+) channels. Endocrinology.

[61] Gibson GE, Manger T, Toral-Barza L, Freeman G (1989). Cytosolic-free calcium and neurotransmitter release with decreased availability of glucose or oxygen. Neurochem Res.

[62] Vidal B, Pasqualini C, Le Belle N, Holland MC, Sbaihi M, Vernier P, Zohar Y, Dufour S (2004). Dopamine inhibits luteinizing hormone synthesis and release in the juvenile European eel: a neuroendocrine lock for the onset of puberty. Biol Reprod.

[63] Simavli S, Thompson IR, Maguire CA, Gill JC, Carroll RS, Wolfe A, Kaiser UB, Navarro VM (2015). Substance p regulates puberty onset and fertility in the female mouse. Endocrinology.

[64] Holesh JE, Bass AN, Lord M: Physiology, Ovulation. In: *StatPearls.* Treasure Island (FL); 2021. 28723025

[65] Lonergan P, Fair T (2016). Maturation of Oocytes in Vitro. Annu Rev Anim Biosci.

[66] Norris RP, Ratzan WJ, Freudzon M, Mehlmann LM, Krall J, Movsesian MA, Wang H, Ke H, Nikolaev VO, Jaffe LA (2009). Cyclic GMP from the surrounding somatic cells regulates cyclic AMP and meiosis in the mouse oocyte. Development.

[67] Vaccari S, Weeks JL, Hsieh M, Menniti FS, Conti M (2009). Cyclic GMP signaling is involved in the luteinizing hormone-dependent meiotic maturation of mouse oocytes. Biol Reprod.

[68] Houslay MD, Griffiths SL, Horton YM, Livingstone C, Lobban M, Macdonald F, Morris N, Pryde J, Scotland G, Shakur Y (1992). Regulation of intracellular cyclic AMP concentrations in hepatocytes involves the integrated activation and desensitization of adenylyl cyclase coupled with the action and activation of specific isoforms of cyclic AMP phosphodiesterase. Biochem Soc Trans.

[69] Olsiewski PJ, Beers WH (1983). cAMP synthesis in the rat oocyte. Dev Biol.

[70] Gilman AG (1990). Regulation of adenylyl cyclase by G proteins. Adv Second Messenger Phosphoprotein Res.

[71] Bernal-Ulloa SM, Heinzmann J, Herrmann D, Hadeler KG, Aldag P, Winkler S, Pache D, Baulain U, Lucas-Hahn A, Niemann H (2016). Cyclic AMP Affects Oocyte Maturation and Embryo Development in Prepubertal and Adult Cattle. PLoS ONE.

[72] Han D, Cao XY, Wang HL, Li JJ, Wang YB, Tan JH (2010). Effects of puberty and gonadotropins on the molecular events controlling meiotic resumption of mouse oocytes. Reproduction.

[73] Shevtsov M, Huile G, Multhoff G: Membrane heat shock protein 70: a theranostic target for cancer therapy. Philos Trans R Soc Lond B Biol Sci 2018, 373(1738). 10.1098/rstb.2016.0526 PMC5717526 29203711

[74] Malami I, Abdul AB (2019). Involvement of the uridine cytidine kinase 2 enzyme in cancer cell death: A molecular crosstalk between the enzyme and cellular apoptosis induction. Biomed Pharmacother.

[75] Michowski W, Chick JM, Chu C, Kolodziejczyk A, Wang Y, Suski JM, Abraham B, Anders L, Day D, Dunkl LM *et al*: Cdk1 Controls Global Epigenetic Landscape in Embryonic Stem Cells. Mol Cell 2020, 78(3):459–476 e413. 10.1016/j.molcel.2020.03.010 PMC7214218 32240602

[76] Endicott JA, Noble ME (1998). Structural principles in cell-cycle control: beyond the CDKs. Structure.

[77] Malumbres M, Barbacid M (2005). Mammalian cyclin-dependent kinases. Trends Biochem Sci.

[78] Satyanarayana A, Kaldis P (2009). Mammalian cell-cycle regulation: several Cdks, numerous cyclins and diverse compensatory mechanisms. Oncogene.

[79] Santamaria D, Barriere C, Cerqueira A, Hunt S, Tardy C, Newton K, Caceres JF, Dubus P, Malumbres M, Barbacid M (2007). Cdk1 is sufficient to drive the mammalian cell cycle. Nature.

[80] Diril MK, Ratnacaram CK, Padmakumar VC, Du T, Wasser M, Coppola V, Tessarollo L, Kaldis P (2012). Cyclin-dependent kinase 1 (Cdk1) is essential for cell division and suppression of DNA re-replication but not for liver regeneration. Proc Natl Acad Sci U S A.

[81] Adhikari D, Zheng W, Shen Y, Gorre N, Ning Y, Halet G, Kaldis P, Liu K (2012). Cdk1, but not Cdk2, is the sole Cdk that is essential and sufficient to drive resumption of meiosis in mouse oocytes. Hum Mol Genet.

[82] Kim J, Singh AK, Takata Y, Lin K, Shen J, Lu Y, Kerenyi MA, Orkin SH, Chen T (2015). LSD1 is essential for oocyte meiotic progression by regulating CDC25B expression in mice. Nat Commun.

[83] Dantas A, Siqueira ER, Fernandes S, Oba E, Castilho AM, Meirelles PRL, Sartori MMP, Santos PTR (2016). Influence of feeding differentiation on the age at onset of puberty in Brazilian Bergamasca dairy ewe lambs. Arq Bras Med Vet Zootec.

[84] Wen B, Zhou R, Feng Q, Wang Q, Wang J, Liu S (2014). IQuant: an automated pipeline for quantitative proteomics based upon isobaric tags. Proteomics.

[85] Brosch M, Yu L, Hubbard T, Choudhary J (2009). Accurate and sensitive peptide identification with Mascot Percolator. J Proteome Res.

[86] Savitski MM, Wilhelm M, Hahne H, Kuster B, Bantscheff M (2015). A Scalable Approach for Protein False Discovery Rate Estimation in Large Proteomic Data Sets. Mol Cell Proteomics.

[87] Zhang Z, Murtagh F, Van Poucke S, Lin S, Lan P (2017). Hierarchical cluster analysis in clinical research with heterogeneous study population: highlighting its visualization with R. Ann Transl Med.

[88] Saldanha AJ (2004). Java Treeview–extensible visualization of microarray data. Bioinformatics.

[89] Ashburner M, Ball CA, Blake JA, Botstein D, Butler H, Cherry JM, Davis AP, Dolinski K, Dwight SS, Eppig JT (2000). Gene ontology: tool for the unification of biology. The Gene Ontology Consortium Nat Genet.

[90] Chen L, Zhang YH, Wang S, Zhang Y, Huang T, Cai YD (2017). Prediction and analysis of essential genes using the enrichments of gene ontology and KEGG pathways. PLoS ONE.

[91] Szklarczyk D, Gable AL, Nastou KC, Lyon D, Kirsch R, Pyysalo S, Doncheva NT, Legeay M, Fang T, Bork P (2021). The STRING database in 2021: customizable protein-protein networks, and functional characterization of user-uploaded gene/measurement sets. Nucleic Acids Res.

[92] Kohl M, Wiese S, Warscheid B (2011). Cytoscape: software for visualization and analysis of biological networks. Methods Mol Biol.

[93] Perez-Riverol Y, Bai J, Bandla C, Garcia-Seisdedos D, Hewapathirana S, Kamatchinathan S, Kundu DJ, Prakash A, Frericks-Zipper A, Eisenacher M (2022). The PRIDE database resources in 2022: a hub for mass spectrometry-based proteomics evidences. Nucleic Acids Res.

