# Supplementary material for: Quantitative proteomics analysis to assess protein expression levels in the ovaries of pubescent goats
Source: BMC Genomics. 2022 Jul 13;23:507. doi: 10.1186/s12864-022-08699-y (PMC9281040; doi:10.1186/s12864-022-08699-y)

Additional file 5. Full-length blot of IGF1 protein expression level. The long blue line indicates the position of the crop.
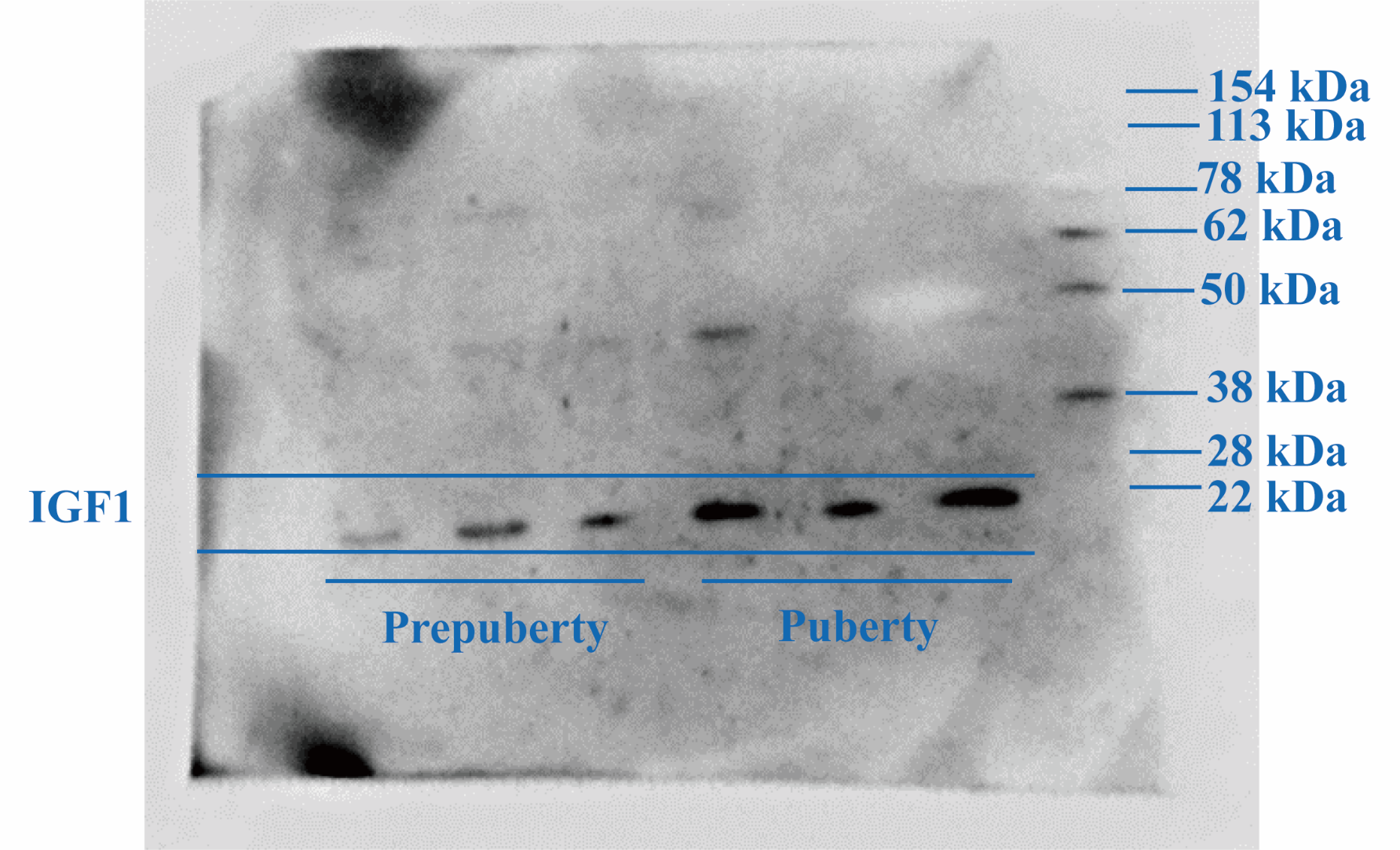


Additional file 6. Full-length blot of RCAN1 protein expression level. The long blue line indicates the position of the crop.
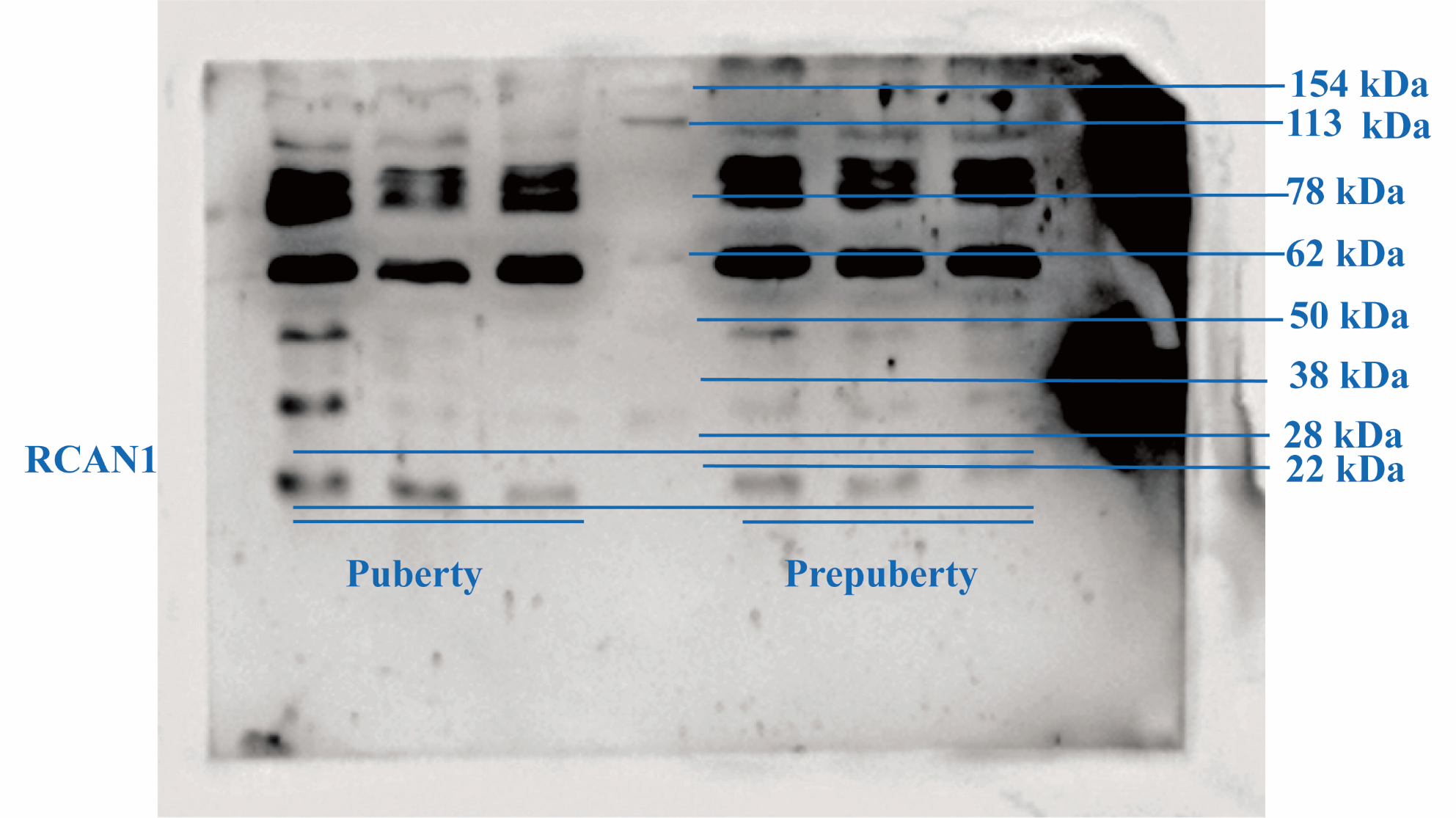


Additional file 7. Full-length blot of DHCR24 protein expression level. The long blue line indicates the position of the crop.
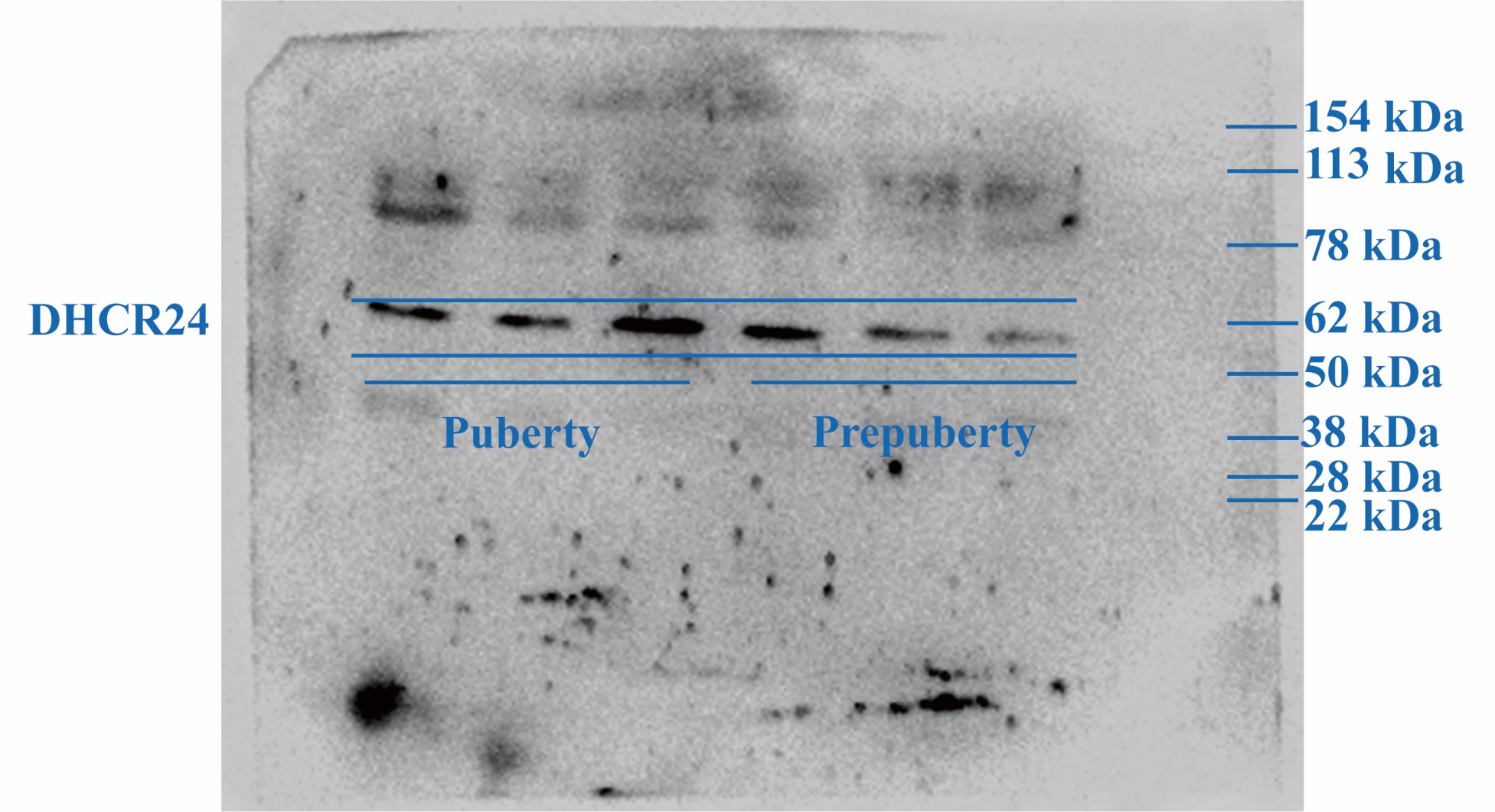


Additional file 8. Full-length blot of CDK1 and β-Tubulin proteins expression level. The long blue line indicates the position of the crop.
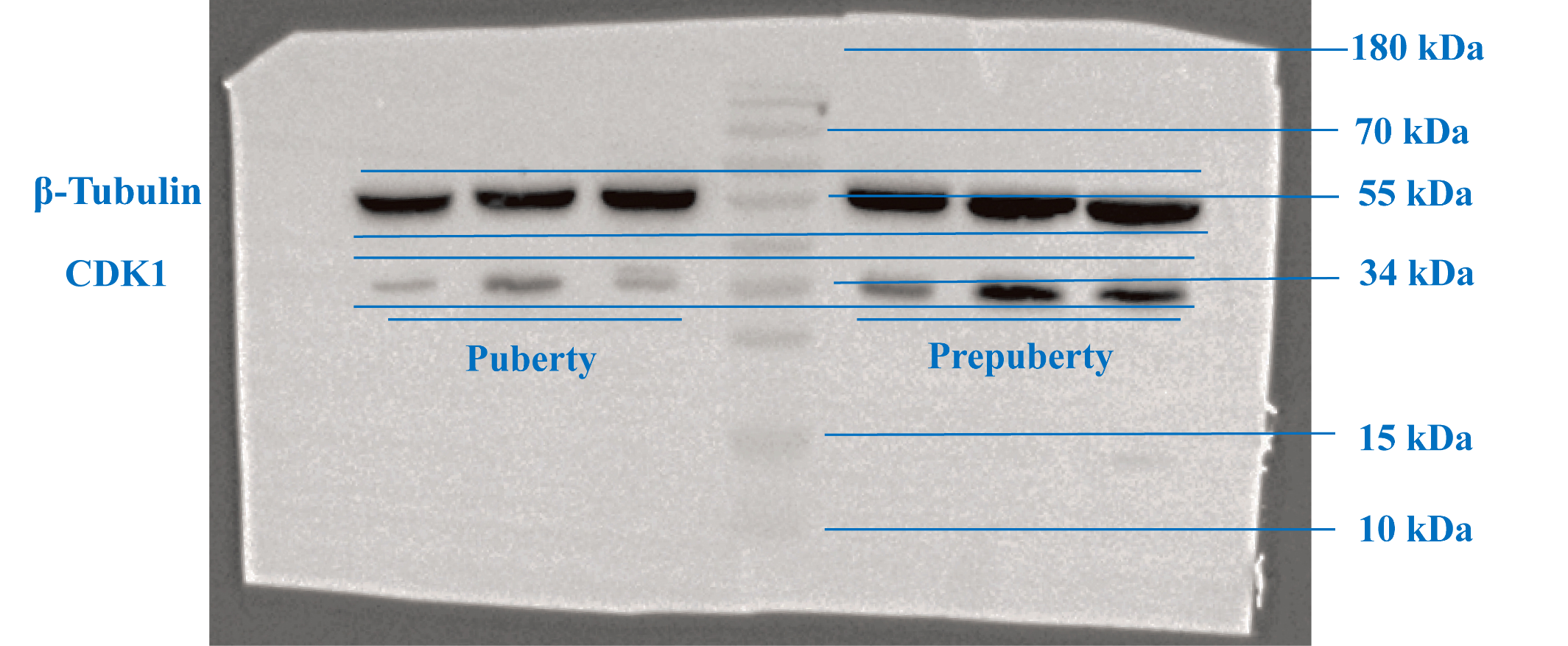


Additional file 9. Full-length blot of STMN1 protein expression level. The long blue line indicates the position of the crop.
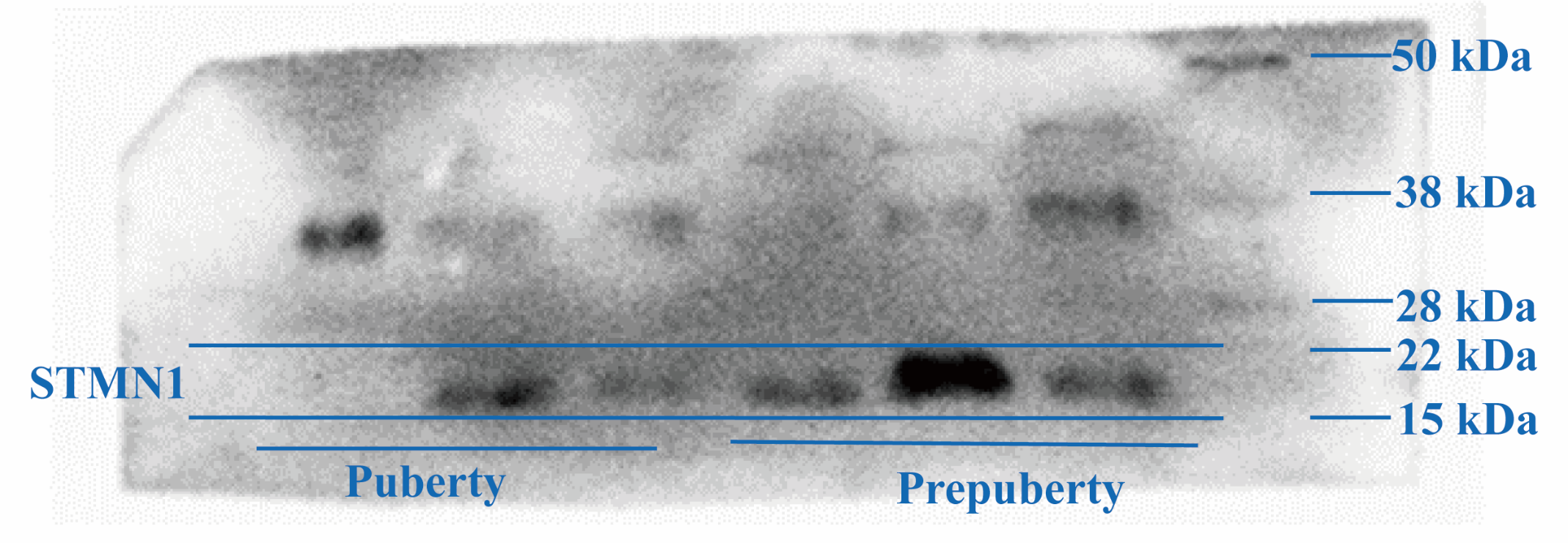

Supplement: Supplementary file 5 — Additional file 5. [file 12864_2022_8699_MOESM5_ESM.docx]
